# Incerto-thalamic modulation of fear via GABA and dopamine

**DOI:** 10.1038/s41386-021-01006-5

**Published:** 2021-04-16

**Authors:** Archana Venkataraman, Sarah C. Hunter, Maria Dhinojwala, Diana Ghebrezadik, JiDong Guo, Kiyoshi Inoue, Larry J. Young, Brian George Dias

**Affiliations:** 1 Emory University Neuroscience Graduate Program, Atlanta, GA USA; 2 Division of Behavioral Neuroscience and Psychiatric Disorders, Yerkes National Primate Research Center, Atlanta, GA USA; 3 Emory University Neuroscience & Behavioral Biology Undergraduate Program, Atlanta, GA USA; 4 Agnes Scott College, Decatur, GA USA; 5 Silvio O. Conte Center for Oxytocin and Social Cognition, Center for Translational Social Neuroscience, Emory University, Atlanta, GA USA; 6 Department of Psychiatry and Behavioral Sciences, Emory University School of Medicine, Atlanta, GA USA; 7 Department of Pediatrics, Keck School of Medicine of USC, Los Angeles, CA USA; 8 Division of Research on Children, Youth & Families, Children’s Hospital Los Angeles, Los Angeles, CA USA; 9 Developmental Neuroscience and Neurogenetics Program, The Saban Research Institute, Los Angeles, CA USA

**Keywords:** Classical conditioning, Behavioural methods

## Abstract

Fear generalization and deficits in extinction learning are debilitating dimensions of Post-Traumatic Stress Disorder (PTSD). Most understanding of the neurobiology underlying these dimensions comes from studies of cortical and limbic brain regions. While thalamic and subthalamic regions have been implicated in modulating fear, the potential for incerto-thalamic pathways to suppress fear generalization and rescue deficits in extinction recall remains unexplored. We first used patch-clamp electrophysiology to examine functional connections between the subthalamic zona incerta and thalamic reuniens (RE). Optogenetic stimulation of GABAergic ZI → RE cell terminals in vitro induced inhibitory post-synaptic currents (IPSCs) in the RE. We then combined high-intensity discriminative auditory fear conditioning with cell-type-specific and projection-specific optogenetics in mice to assess functional roles of GABAergic ZI → RE cell projections in modulating fear generalization and extinction recall. In addition, we used a similar approach to test the possibility of fear generalization and extinction recall being modulated by a smaller subset of GABAergic ZI → RE cells, the A13 dopaminergic cell population. Optogenetic stimulation of GABAergic ZI → RE cell terminals attenuated fear generalization and enhanced extinction recall. In contrast, optogenetic stimulation of dopaminergic ZI → RE cell terminals had no effect on fear generalization but enhanced extinction recall in a dopamine receptor D1-dependent manner. Our findings shed new light on the neuroanatomy and neurochemistry of ZI-located cells that contribute to adaptive fear by increasing the precision and extinction of learned associations. In so doing, these data reveal novel neuroanatomical substrates that could be therapeutically targeted for treatment of PTSD.

## Introduction

The expression of maladaptive fear is a core pathology of Post-Traumatic Stress Disorder (PTSD) [1–5]. Fear of trauma-associated stimuli even after they have ceased to be threats is one such highly prevalent and debilitating form of maladaptive fear. Such fear results from being unable to *learn* that stimuli previously linked to trauma are no longer dangerous (deficits in extinction learning). Another form of maladaptive fear that is a highly prevalent dimension of PTSD is fear generalization—fear expressed toward neutral stimuli that resemble trauma-associated stimuli but that were not directly associated with threat. Understanding the neural circuitry that modulates generalization and extinction of traumatic fear memories can help us design effective therapeutic strategies to reduce the debilitating expression of fear accompanying PTSD. Studies with this intent have thus far largely focused on examining canonical fear-related circuitry like the amygdala, prefrontal cortex, and hippocampus [6–12]. In contrast, very little is understood about the role of thalamic and subthalamic regions in modulating fear generalization and extinction learning. Thalamic and subthalamic brain regions have traditionally been considered merely as hubs that relay information from sensory cortices to limbic, midbrain, and brainstem nuclei. However, emerging literature suggests that several of these non-canonical brain regions contribute to fear-related behaviors [13–17].

A growing body of research implicates the subthalamic zona incerta in modulating fear [18–21]. As a key site of sensorimotor integration, the ZI has access to sensory information and co-ordinates behavioral responses through its efferent projections. Activation of the ZI has been shown to suppress generalization and enhance extinction of fear memories [18, 19]. More specifically, stimulation of GABAergic cells in the ZI have been shown to suppress fear generalization and enhance extinction learning. However, it is unclear which GABAergic projections from the ZI influence fear generalization and extinction learning. In addition, the GABAergic cells in the ZI co-express other neuromodulators including dopamine and somatostatin [21–23], raising the possibility of differential modulation of fear based on their neurochemical profile. Given the documented contributions of A10 dopaminergic cells in the ventral tegmental area (VTA) to fear generalization and extinction learning [9, 24–26], we sought to delineate the functional contributions of ZI-located GABAergic cells and a smaller subset of these GABAergic cells, the A13 dopaminergic cells, to these processes.

Amidst the extensive connectivity of the ZI, we were struck by its dense projections to the thalamic nucleus reuniens (RE) [19]. The RE serves as a critical hub connecting the medial prefrontal cortex to the hippocampus and plays a pivotal role in emotional regulation [16, 17, 27–30]. Importantly, the RE is required for the maintenance of precise fear memory associations, thereby controlling the extent of generalization and extinction [16, 17, 29, 31]. Despite this well-established connectivity of the ZI with the RE and the important role that the RE plays in both, fear generalization and extinction, the ability of ZI → RE projections to rescue deficits in fear inhibition have yet to be explored. Based on the anatomical observations from our studies and others, and the established functions of RE, we hypothesized a modulatory role for GABAergic and dopaminergic ZI → RE cells in rescuing deficits in fear inhibition.

## Materials and methods

### Animals

vGAT-CRE and TH-CRE mice were acquired from Jackson labs and then bred in the vivarium with controlled temperature, humidity, and pressure. Adult female and male vGAT-CRE and TH- CRE mice (2–3 months of age) were group-housed and kept on a 14:10 light/dark cycle with *ad libitum* access to standard chow and water. Our previously published data did not uncover any sex differences in the modulation of fear generalization and extinction learning by GABAergic cells in the ZI [19]. Similarly, we do not observe sex differences in the results that we report and show data split by sex in the Supplementary Material. All experimental procedures were performed during the light cycle and were approved by the Emory Institutional Animal Care and Use Committee, in accordance with National Institute of Health guidelines.

### Virus injection and fiber optic implantation

For optogenetic stimulation of the ZI-RE pathway, vGAT-CRE mice (targeting GABAergic cells) or TH-CRE mice (targeting A13 dopaminergic cells) were injected with AAV5-EF1α -DIO-mCherry or AAV5-EF1α-DIO-ChR2(H134R)-mCherry obtained from the University of North Carolina Viral Vector Core.

Animals were anesthetized with ketamine/dexdomitor i.p. injection and placed in a stereotaxic device for virus placement and optic fiber insertion. AAV constructs were bilaterally injected using Nanoject III (Drummond Scientific) into the ZI of vGAT-CRE mice using the following stereotaxic coordinates: AP: −1.52 mm, ML: ± 0.73 mm, and DV: −4.79 mm relative to Bregma. A13 cells within the ZI were targeted in TH-CRE mice using the following stereotaxic coordinates: AP: −1.40 mm, ML: ± 0.68 mm and DV: −4.79 mm relative to Bregma. The total volume of AAV-containing solutions injected into the ZI was 150 nl per side, at the rate of 1 nl/s. After injection, the needle was left in place for an additional 10 min and slowly withdrawn over 1 min. Mice were allowed to recover for at least 6 weeks to allow for optimal expression of the opsin. To stimulate the ZI GABAergic and dopaminergic cell terminals in the RE, animals were anesthetized again and implanted with the fiber optic cannula (200 µm diameter, NA 0.39, Thorlabs) at midline position above the RE at AP: −0.38 mm, ML: 0 mm and DV: −4.5 mm relative to Bregma. Mice were allowed to recover for 1 week and then handled for 5 min each day for 5 days before the start of behavioral experiments.

### Behavioral procedures

Behavioral sessions were conducted in conditioning chambers (Coulbourn Instruments) connected to a tone generator. The high-intensity auditory fear conditioning procedure was used to induce fear generalization and deficits in extinction learning as previously described in [7, 19, 32–34].

#### Cue-dependent fear conditioning

Briefly, mice were pre-exposed to context A for 5 min, once daily for 2 days before training. On training day, following a 5-min exposure to context A, mice received 10 paired CS+ tone presentations (30 s, 75–80 dB) that co-terminated with high-intensity foot-shocks (0.5 s, 0.8 mA) alternating pseudo-randomly with 10 unpaired CS− presentations (30 s, 80–85 dB). The inter-trial intervals (ITIs) were set to vary between 2 and 6 min. Conditioning sessions were conducted in Context A illuminated with house lights and consisted of grid floor cleaned with quatricide disinfectant.

#### Testing fear generalization and extinction training

The next day, during the testing session, mice were exposed to context B for 3 min. Context B consisted of the house lights being off and plexiglass flooring cleaned with 70% EtOH. Infrared lights in the chambers allowed for the cameras to record behavior. In the vGAT-CRE experiments (outlined in Fig. 2A, B), animals received two randomized presentations of the CS+ and CS− tones (30 s, 80–85 dB, 3 min ITI) with no laser stimulation (‘laser OFF’ condition) and two randomized presentations of the CS+ and CS− tones (30 s, 80–85 dB, 3 min ITI) in the ‘laser stim’ condition (laser stimulation was applied for the 30 secs of the CS+ and CS− presentation with details noted below). While remaining in the same chambers, the mice were immediately given extinction training during which they received 28 presentations of the CS+ tone (30 s, 80–85 dB, 30 s ITI) with each CS+ presentation accompanied by optogenetic stimulation (laser stimulation was applied for the 30 s of the CS+ presentations with details noted below). One randomized 30-s trial with laser stimulation alone (in the absence of tone) was presented to assess non-specific behavioral effects.

In the TH-CRE experiments (outlined in Fig. 4A, B), animals received i.p. injection of VEH or selective DRD1 antagonist SCH 23390 (0.1 mg/kg in 0.9% saline) prior to the start of the session. During the testing session, mice received two randomized presentations of the CS+ and CS− tones (30 s, 80–85 dB, 3 min ITI) in context B to assess fear generalization. Following this, animals were immediately trained to extinguish fear with exposure to 28 CS+ tone presentations (30 s, 80–85 dB, 30 s ITI) in the absence of shock, while remaining in the same chambers. All tone presentations were paired with optogenetic stimulation (‘laser stim’ condition). One randomized 30-s trial with laser stimulation alone was presented to assess any non-specific behavioral effects.

#### Testing recall of extinction learning

Twenty-four hours after the extinction training, mice were exposed to context B for 2 min. Mice then received 2 presentations of CS+ tones (30 s, 80–85 dB, 30 s ITI) in the absence of any optogenetic stimulation.

#### Behavioral analyses

All behavioral sessions were video recorded and freezing behavior was analyzed using FreezeFrame-4 software (Actimetrics). The total amount of time spent freezing (in seconds) to the tones, context, or laser stimulation alone was analyzed in 30-s bins using FreezeFrame software by an experimenter blind to the treatment conditions.

### Optogenetic stimulation

The implants placed above the RE consisted of Ø200 µm optic fiber (NA = 0.39, Thorlabs) held in a ceramic ferrule (1.25 mm, Thorlabs). The optic fibers were cut and polished to a length of 5 mm from the bottom of the ferrules, so as to reach the RE. The optic fibers were connected to patch cables (Ø200 µm, NA = 0.22) that were in turn connected to a laser light source (473-nm lasers, DPSS Systems, Shanghai Laser & Optics Century). For optogenetic stimulation in the ‘laser stim’ conditions, pulses of blue laser light were delivered at a frequency of 20 Hz (in vGAT-CRE animals) and 10 Hz (in TH-CRE animals). The blue laser pulses were delivered for the entire 30-s period during the CS+ and CS− tone presentations.

### Slice preparation and electrophysiological recording

To confirm ChR2 function and determine the optimal stimulation paradigm, vGAT-CRE, and TH-CRE mice injected with the ChR2 viruses were sacrificed 8–10 weeks later and electrophysiological recordings were performed as previously reported in [35]. Briefly, 300-µm-thick coronal brain slices containing the ZI and/or RE were prepared using a VTS-1000 vibrating blade microtome (Leica Microsystems Inc., Bannockburn, IL, USA) from adult vGAT-CRE and TH-CRE mice anesthetized with isoflurane before decapitation. Brain slices were removed and placed in 95% oxygen-5% carbon dioxide oxygenated artificial cerebrospinal fluid (ACSF) at 32 °C for 1 h before transfer to the recording chamber mounted on the stage of Leica STP6000 microscope. The slices were completely submerged and continuously perfused with oxygenated ACSF at 32 °C at a speed of ~2 ml/min.

Brain regions were located using differential interference contrast optics and an infrared-sensitive CCD camera (Orca ER, Hamamatsu, Tokyo, Japan). GABAergic or A13 dopaminergic ZI neurons and their projections were visually identified in vGAT-CRE and TH-CRE mice, respectively, by the expression of mCherry fluorescent transgene using an epifluorescence microscope. Patch pipettes were pulled from thin-walled borosilicate glass capillary tubes and filled with a solution made up of the following components (in mM): 130 K-Gluconate, 2 KCl, 10 HEPES, 3 MgCl_2_, 5 phosphocreatine, 2 K-ATP, and 0.2 NaGTP, buffered to a pH of 7.3 and an osmolarity of 280–290 mOsm. The resistance of the pipettes varied between 4 and 6 MΩ. The current and voltage signals were recorded using a MultiClamp 700B amplifier, in conjunction with an Axon Digidata 1550 A–D interface and pClamp 10.4 software (Molecular Devices, Sunnyvale, CA).

#### Light stimulation of ZI neurons

Laser illumination (wavelength 473 nm, 2–5 mW mm^−2^) was delivered through an optic fiber positioned above the brain tissue connected to a solid-state laser (Shanghai Laser & Optics Century) and oriented directly toward the recorded neurons. Single light pulses were delivered at increasing intensities to obtain intensity–response curve and threshold for action potentials. Stimulus trains of 1-s light pulses (1 ms pulse width, frequencies of 10, 20, 30, and 50 Hz) at 1.0–1.2-fold of the spike threshold were delivered to induce spike trains. In coronal slices containing both ZI and RE, a laser fiber was placed above RE neurons surrounded by mCherry positive fibers to characterize the ZI-RE pathway. The effect of bath application of AMPA/kainite receptor antagonist 6,7-dinitroquinoxaline-2,3-dione (DNQX) and GABA-A receptor antagonist gabazine on light-evoked IPSCs, was examined. All drugs were purchased from Tocris and applied by gravity perfusion in the circulating ACSF medium.

### Histology and RNAScope in situ hybridization

vGAT-CRE and TH-CRE mice were trans-cardially perfused with 4% paraformaldehyde (PFA) dissolved in 1× phosphate-buffered saline. Brains were harvested and fixed in 4% PFA for a day and equilibrated in 30% sucrose solution for 3–4 days. Brains were sectioned at 35 μm on a freezing microtome (Leica). For verification of virus expression and optic fiber placement, the 35-μm sections were stained with Hoechst nuclear stain (1:1000) and mounted on slides using SlowFade Gold Antifade mountant (Life Technologies). The position of the mCherry positive cells was assessed using a Nikon Eclipse E800 fluorescent microscope.

Fluorescent triple-labeling in situ hybridization was performed using the RNAscope Fluorescent Multiplex Reagent Kit (Advanced Cell Diagnostics) according to the manufacturer’s instructions. Briefly, 14-μm-thick fresh frozen brain sections were thawed and fixed in 10% neutral buffered formalin solution for 15 min. After treatment with RNAscope Protease IV for 30 min, sections were hybridized with mouse TH, vGAT, and vGlut2-specific probes (Cat. No.317621, 319191 and 319171, respectively) at 40 °C for 2 h. After three steps of amplification, hybridized probes with TH, vGAT, and vGlut2 mRNA were labeled and visualized with Alexa 488, Atto 550, and Atto 647 fluorescent dyes, respectively. Nuclei were visualized with DAPI and sections were mounted with ProLong gold antifade mountant (Thermo Fisher Scientific). Negative control with the DapB probe (Cat No. 310043) did not show any non-specific signal, which was similar to negative control without probes. Fluorescent images were captured with z-stack function using a BZ-X710 Keyence microscope.

### Statistical analysis

Statistical data analyses were performed using GraphPad Prism. Single-variable differences in data sets containing only two groups were analyzed using unpaired *t*-tests. Group differences were analyzed using two-way repeated measures ANOVA where appropriate. Significant interactions in the ANOVAs were analyzed for multiple comparisons using the Holm–Sidak test. For all analyses, the significance level was set at *p* < 0.05.

## Results

### Selective optogenetic targeting and functional validation of GABAergic projections from ZI to RE

**Fig. 1:**
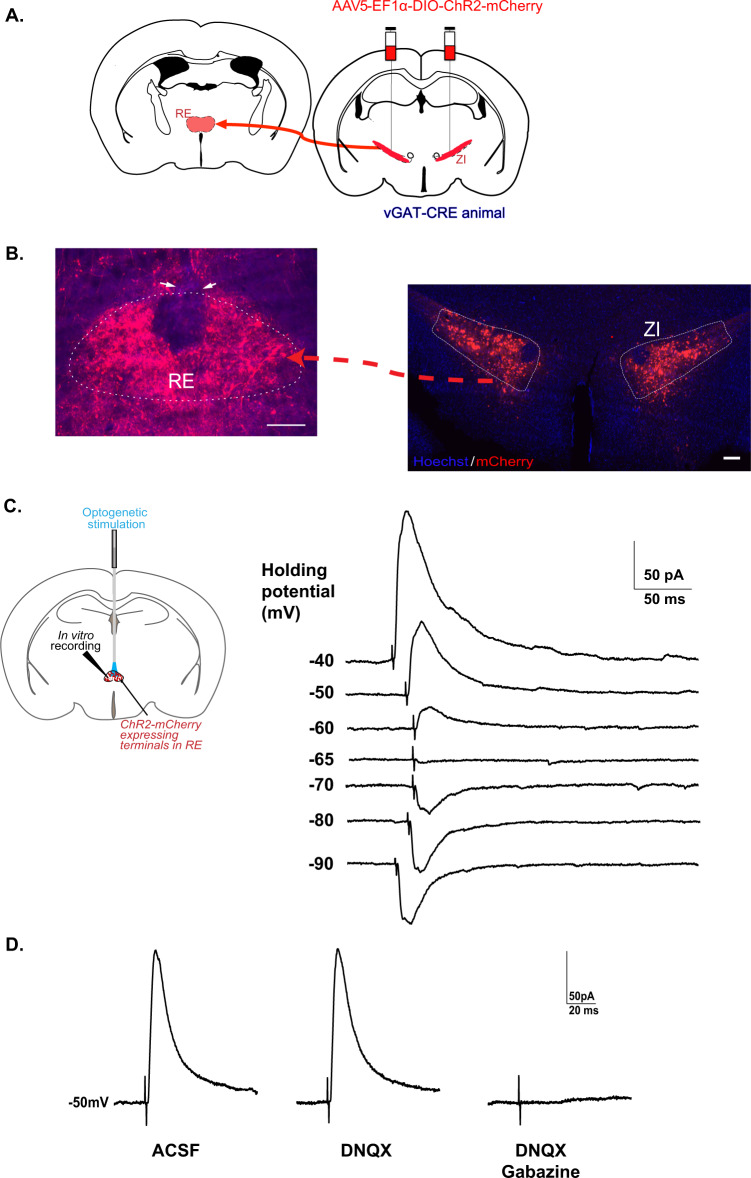
Optogenetic stimulation of GABAergic cells in ZI-induced IPSCs in RE neurons. **A** vGAT-CRE mice were injected with Cre-dependent ChannelRhodopsin2 (AAV5-EF1α-DIO-ChR2-mCherry) at −1.5 mm posterior to bregma and the optic fiber was placed above the RE at −0.38 mm posterior to bregma. **B** Representative image of the ZI targeted with intracranial infusions of ChR2-expressing mCherry viruses (right) and representative image of the mCherry expressing GABAergic projection fibers in the RE with cannula placed above the region (indicated by arrows) (left). **C** Illustration of experimental configuration for optic stimulation and in vitro patch-clamp recordings from RE neurons (left). Optic stimulation (473 nm blue light, 1.6 mW/mm^2^) was directed at ChR2-expressing GABAergic projections in RE. Sample traces of superimposed light-evoked currents recorded at the indicated holding potentials shown to the left of each trace (right). The evoked inhibitory post-synaptic currents (IPSCs) had a reversal potential of −65 mV, close to chloride equilibrium potential. **D** Bath application of 20 µM AMPA receptor antagonist DNQX had no effect on light-evoked IPSCs while 5 µM of GABA receptor antagonist gabazine completely blocked light-evoked IPSCs. Scale bars: 100 µm.

To identify GABAergic projections from the ZI → RE, we injected a CRE-dependent adeno-associated viral vector expressing ChR2-mCherry into the ZI of vGAT-CRE mice (as outlined in Fig. 1A and directly visualized in Fig. 1B). This allows expression of the stimulatory opsin ChR2 in the GABAergic cells of the ZI and more importantly, in their terminals in regions directly innervated by these GABAergic cells. Four to six weeks later, we found dense ChR2-mCherry expression in the GABAergic cell bodies (Fig. 1B, Supplementary Fig. 1A) in the ZI and terminals that innervate the RE (Fig. 1B, Supplementary Fig. 1B). To further confirm the function of the stimulatory opsin ChR2, we performed whole-cell patch-clamp recordings on ChR2-mCherry expressing GABAergic neurons in the ZI (Supplementary Fig. 2A–E). Next, to validate the functional connectivity between GABAergic cells in the ZI and thalamic RE, we performed whole-cell patch-clamp recordings from RE neurons. Optical stimulation (473-nm-blue light, 1.6 mW/mm^2^) of ChR2-mCherry expressing GABAergic projection fibers originating from the ZI, induced inhibitory post-synaptic currents (IPSCs) in RE (Fig. 1C). The evoked IPSCs had a reversal potential of ~65 mV, which is close to chloride equilibrium potential. Blocking glutamatergic transmission with AMPA receptor antagonist DNQX (20 µM) had no effect on the evoked IPSCs. Next, when we recorded in the presence of both DNQX (20 µM) and GABA antagonist gabazine (5 µM) the IPSCs were completely abolished (Fig. 1D). These results confirmed the presence of a distinct inhibitory pathway from subthalamic ZI to thalamic RE.

### Optogenetic stimulation of ZI → RE GABAergic projections reduces fear generalization

**Fig. 2:**
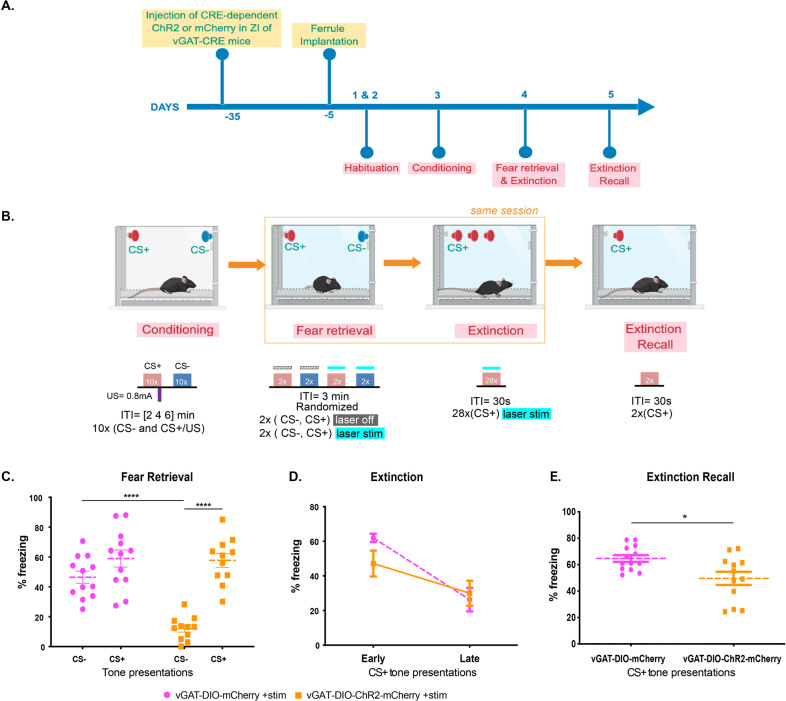
Targeted optogenetic stimulation of ZI-RE GABAergic projections reduces fear generalization and enhances extinction recall. **A** Experimental design: vGAT-CRE animals received intracranial injections of CRE-dependent control or ChR2 (ChannelRhodopsin2) virus in the ZI and after 4 weeks, were implanted with fiber optic cannula in the RE. After recovery from the implantation surgeries, animals were first habituated and then fear conditioned to tones using high shock intensities. After 24 h, animals were tested for fear generalization and went through extinction training in the same session. The following day, animals were tested for extinction recall. **B** Outline of the high-intensity auditory fear conditioning protocol used in the study. On training day, both control and treatment groups of mice received CS+ tone presentations paired with 0.8 mA foot-shocks (high threat intensity) and unpaired CS− tone presentations. On testing day, freezing responses in both groups of animals were recorded for the CS+ and CS− tone presentations without laser stimulation and with laser stimulation. Immediately following the testing session and without removing animals from the chambers, animals received repeated presentations of CS+ tones as part of the extinction session paired with laser stimulation. Laser stimulation when applied, was done so for the entire 30-s duration of each CS+ and CS− presentation. One day later, fear responses of the animals to the CS+ tones were tested. **C** Optogenetic stimulation of ZI-RE GABAergic projections (vGAT-DIO-ChR2-mCherry + stim) during fear retrieval resulted in a significant decrease in fear response to CS− compared to controls (vGAT-DIO-mCherry + stim). **D** Optogenetic activation of ZI-RE GABAergic projections during extinction learning did not produce any significant differences in freezing responses between the two groups. **E** During extinction recall, animals that previously received optogenetic stimulation of ZI-RE GABAergic projections (vGAT-DIO-ChR2-mCherry) showed a significant decrease in fear response to CS+ compared to controls (vGAT-DIO-mCherry) *p < 0.05, ****p < 0.0001. Data represented as mean ± SEM.

To determine whether stimulation of GABAergic projections from the ZI to RE affects fear generalization, we injected AAV5-EF1α-DIO-ChR2-mCherry (stimulatory opsin) or AAV5-EF1α-DIO-mCherry bilaterally into the ZI of vGAT-CRE mice followed by optic fiber implantation in the RE. The mice were then trained in a high-intensity discriminative auditory fear conditioning protocol (as described in Fig. 2A, B) that has been shown to produce generalization of fear associations to both the aversive CS+ as well as the neutral CS− tones [7, 19, 33]. One day after training, mice were tested for fear generalization with the laser light pulsed during the CS+ and CS− presentations (Fig. 2B). Increased fear generalization was observed after optical stimulation of RE in vGAT-CRE animals that received control DIO-mCherry virus. However, increasing activity of GABAergic projections in the RE originating from the ZI with laser stimulation robustly reduced fear generalization (Fig. 2C). Specifically, vGAT-CRE: DIO-ChR2-mCherry+stim animals exhibited significantly lower freezing responses to CS− compared to vGAT-CRE:DIO-mCherry+stim animals (vGAT-CRE:DIO-mCherry group *n* = 12, vGAT-CRE:DIO- ChR2-mCherry *n* = 11, Virus × Tone interaction: *F*(1,21) = 24.20, *p* < 0.0001. Post-hocs: vGAT-CRE: DIO-ChR2-mCherry+stim:CS+ vs. vGAT-CRE: DIO-ChR2-mCherry+stim:CS− *p* < 0.0001, vGAT-CRE:DIO-mCherry:CS− vs. vGAT-CRE: DIO-ChR2-mCherry+stim:CS− *p* < 0.0001). Further, vGAT-CRE:DIO-ChR2-mCherry+stim showed better discrimination between the CS+ and the CS− tones compared to vGAT-CRE:DIO-mCherry+stim controls as evident from the discrimination index (Supplementary Fig. 3). Notably, in the absence of laser stimulation, both vGAT-CRE:DIO-mCherry controls and vGAT-CRE:DIO-ChR2-mCherry animals showed high levels of freezing to the CS+ and CS− and poor discrimination (Supplementary Fig. 4). Optogenetic stimulation of the ZI-RE GABAergic projections in the absence of tone presentations did not produce any significant difference in freezing levels between the two groups (*t* = 1.56, df = 21, *p* > 0.05) (Supplementary Fig. 5). [vGAT-DIO-mCherry *n* = 12 (6 M, 6 F), vGAT-DIO-ChR2 *n* = 13 (5 M, 8 F)]. We did not observe any sex differences in fear generalization and therefore report our data in Fig. 2C collapsed across sexes [Three-way ANOVA: Tones × Opto × Sex. Tones × Opto × Sex Interaction: *p* = 0.6728, Opto × Sex Interaction: *p* = 0.4986, Tones × Opto Interaction: *p* = 0.0074)]. We have reported our data split by sex in Supplementary Fig. 6.

### Optogenetic stimulation of ZI → RE GABAergic projections enhances extinction recall

To determine whether stimulation of GABAergic projections from the ZI to RE affects fear extinction, all vGAT-CRE animals were trained to extinguish fear responses to CS+ tones in the presence of optogenetic stimulation (as described in Fig. 2A, B). We found no statistically significant difference in fear responses to the CS+ tones between control (vGAT-CRE: DIO-mCherry+stim) and experimental groups (vGAT-CRE: DIO-ChR2+stim) (Fig. 2D). However, one day after this extinction training, vGAT-CRE: DIO-ChR2 animals expressed better extinction recall compared to vGAT-CRE: DIO-mCherry animals. Specifically, vGAT-CRE: DIO-ChR2-mCherry animals exhibited significantly lower freezing responses to CS+ tone presentations compared to vGAT-CRE:DIO-mCherry animals (Fig. 2E) (vGAT-CRE:DIO-mCherry group *n* = 13, vGAT-CRE:DIO- ChR2-mCherry *n* = 12, *p* < 0.05, t = 2.750, df = 23). [vGAT-DIO-mCherry *n* = 12 (6 M, 6 F), vGAT-DIO-ChR2 *n* = 13 (5 M, 8 F)]. We did not find any sex differences in our data and therefore report our data in Fig. 2E collapsed across sexes [Two-way ANOVA: Opto × Sex. Opto × Sex Interaction: *p* = 0.4691, Sex Main effect: *p* = 0.2975, Opto Main Effect: *p* = 0.01)]. We have reported our data split by sex in Supplementary Fig. 7.

### A13 dopaminergic cells in the ZI are GABAergic and project to the RE

**Fig. 3:**
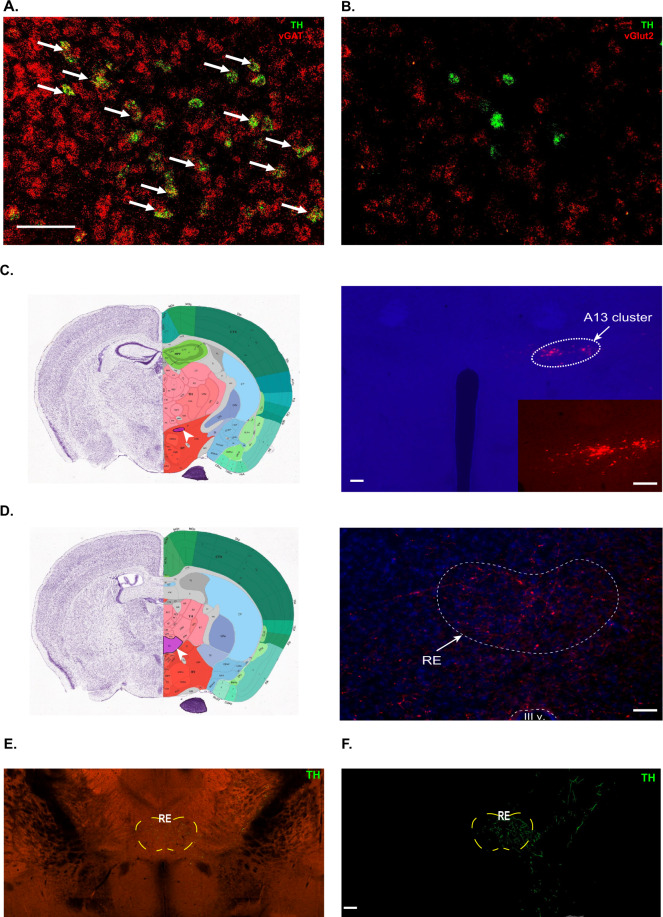
A13 dopaminergic cells co-express GABA and project to the RE. **A**, **B** RNAScope in situ hybridization reveals overlap of *th* (green) and *vgat* mRNA, but not of *th* (green) and *vglut2* mRNA in the ZI. **C**, **D** Unilateral injection of AAV5-DIO-mCherry into the ZI of TH-CRE mice revealed projections in the RE. **E**, **F** Serial two-photon tomography image and projection segmentation image from Allen Mouse Brain Connectivity Atlas showing dopaminergic projections in the RE after injection of DIO-eGFP into A13 cells of the ZI in TH-CREFI172 mice (Experiment 306270474). Scale bars: 100 µm.

With evidence demonstrating that the A13 dopaminergic cell population in ZI are a subset of GABAergic cells in the ZI [23], that A13 cells project to the RE [36, 37], and that dopamine plays a role in fear generalization and extinction learning [24–26], we sought to further characterize the neurochemistry and projections of the A13 cells. First, using RNAScope-based in situ hybridization and confirming prior work, we observed colocalization between TH (marker of dopaminergic cells) and vGAT (marker of GABAergic cells) but not between TH and vGlut2 (marker of glutamatergic cells) (Fig. 3A, B). Next, we examined whether these dopaminergic cells contact the RE. Anterograde tracing revealed dopaminergic cell terminals in the thalamic RE (Fig. 3C, D), as has been demonstrated previously by the Allen Brain Atlas tracing project (Fig. 3E, F). To validate our findings, we also looked at regions that have been reported to be innervated by A13 cells and found for example, that A13 cells project to the paraventricular nucleus of the hypothalamus (Supplementary Fig. 8) as has been previously noted [36, 37]. Importantly, our viral infusions are restricted to the A13 population of dopaminergic cells in the ZI and we do not observe non-specific staining of the A10 (VTA) dopaminergic cells (Supplementary Fig. 9).

### Optogenetic stimulation of A13 ZI → RE dopaminergic terminals does not alter fear generalization but enhances extinction recall via dopamine receptor D1 (DRD1) action

**Fig. 4:**
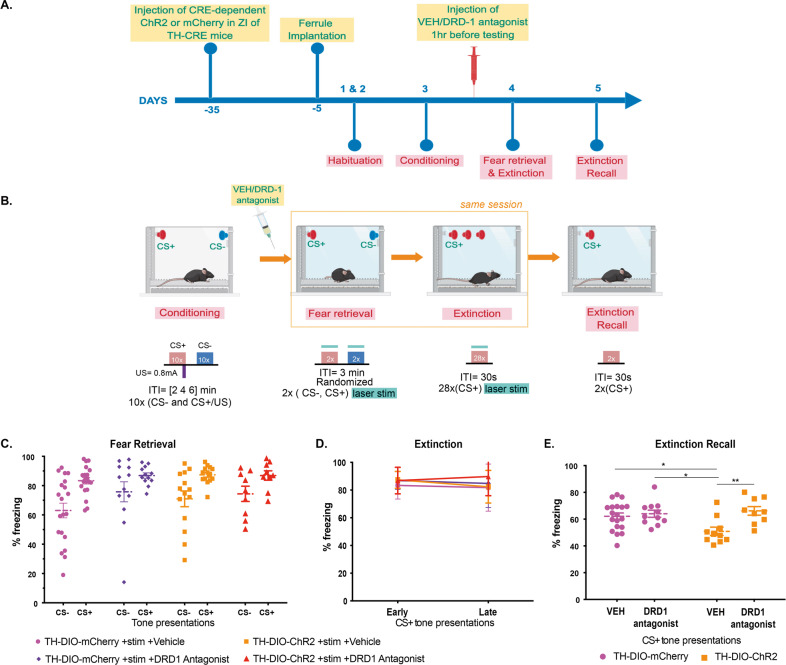
Targeted optogenetic stimulation of ZI-RE dopaminergic projections enhances extinction recall. **A** Experimental design: TH-CRE animals received intracranial injections of CRE-dependent control or ChR2 (ChannelRhodopsin2) virus in the ZI and after 4 weeks, were implanted with fiber optic cannula in the RE. After recovery from the implantation surgeries, animals were first habituated and then fear conditioned to tones using high shock intensities. 24 h later, animals were tested for fear generalization. Immediately after, animals went through an extinction session. The following day, animals were tested for extinction recall. **B** Outline of the high-intensity auditory fear conditioning protocol used in the study. On training day, both control and treatment groups of mice received CS+ tone presentations paired with 0.8 mA foot-shocks (high threat intensity) and unpaired CS− tone presentations. On testing day, freezing responses in both groups of animals were recorded for the CS+ and CS− tone presentations while laser stimulation occurred for the entire 30-s duration of each CS+ and CS− presentation. Immediately following the testing session and without removing animals from the chambers, animals received repeated presentations of CS+ tones as part of the extinction session with laser stimulation occurring for the entire 30-s duration of each CS+ presentation. One day later, fear responses of the animals to the CS+ tones were tested. **C** Animals injected with DIO-ChR2 virus in TH-CRE expressing A13 dopaminergic cells in the ZI and optic cannula in the RE receiving A13 dopaminergic projections (TH-DIO-ChR2-mCherry + stim + Vehicle) showed no significant differences in fear response to CS+ and CS− compared to animals that were infused with the DIO-mCherry virus (TH-DIO-mCherry + stim + Vehicle) in TH-CRE expressing A13 dopaminergic cells in the ZI and optic cannula in the RE receiving A13 dopaminergic projections. Pre-treatment with the DRD1 antagonist (SCH 23390, 0.1 mg/kg, i.p. in 0.9% saline Vehicle) did not affect freezing to the CS+ and CS− (TH-DIO-mCherry + stim + DRD1Antagonist, TH-DIO-ChR2-mCherry + stim + DRD1Antagonist). **D** Optogenetic activation of ZI-RE dopaminergic projections during extinction learning did not produce any significant differences in freezing responses between the four groups. **E** During extinction recall, animals that previously received optogenetic stimulation of ZI-RE dopaminergic projections (TH-DIO-ChR2-mCherry + stim + Vehicle) showed a significant decrease in fear response to CS+ compared to controls (TH-DIO-mCherry + stim + Vehicle). Importantly, pre-treatment with DRD1 antagonist blocked this enhancement of extinction recall (TH-DIO-ChR2-mCherry + stim + Vehicle vs TH-DIO-ChR2-mCherry + stim + DRD1 Antagonist) *p < 0.05, **p < 0.01. Data represented as Mean ± SEM.

To determine the effects of stimulation of dopaminergic projections from the ZI to RE on fear inhibition, we injected AAV5-EF1α-DIO-ChR2-mCherry (stimulatory opsin) (Supplementary Fig. 10) or AAV5-EF1α-DIO-mCherry bilaterally into the ZI of TH-CRE mice followed by optic fiber implantation in the RE. The mice were then trained in a high-intensity auditory fear conditioning protocol (as described in Fig. 4A, B). One day after training, no change in fear generalization was observed in TH-CRE animals that received optogenetic stimulation of RE during presentations of CS− and CS+ tones (Fig. 4C). Further, optogenetic stimulation during the extinction of CS+ tones (TH-CRE: DIO-ChR2) did not produce any significant difference in freezing responses compared to controls (TH-CRE:DIO-mCherry) (Fig. 4D). Similarly, injection of selective DRD1 selective antagonist SCH 23390 (0.1 mg/kg, i.p. in 0.9% saline) before testing did not affect fear generalization or within-session extinction (Fig. 4C, D). All groups show low discrimination between the CS− and CS+ as evident from the discrimination index (Supplementary Fig. 11). Excitingly, stimulation of A13 ZI → RE dopaminergic terminals during extinction training resulted in enhanced extinction recall 24 h later, an effect that was blocked upon pre-treatment with SCH 23390 [Virus x DRD1 status interaction: *F* (1, 45) = 5.108 *p* = 0.0287. Post-hocs: Vehicle:TH-DIO-mCherry+stim vs. Vehicle:TH-DIO-ChR2+stim *p* < 0.05, Vehicle:TH-DIO-ChR2+stim vs. DRD1 Antag:TH-DIO-GFP + stim *p* < 0.05, Vehicle:TH-DIO-ChR2 + stim vs. DRD1 Antag:TH-DIO-ChR2 + stim *p* < 0.01] (Fig. 4E). Optogenetic stimulation of the A13 projection fibers in the RE alone did not produce non-specific effects on freezing levels (ANOVA: F(3,51) = 0.8194, p > 0.05) (Supplementary Fig. 12). [TH-DIO-mCherry + Veh: *n* = 20 (10 M, 10 F), TH-DIO-ChR2 + Veh: *n* = 12, (7 M, 7 F), TH-DIO-mCherry + DRD1 Antagonist: *n* = 11 (6 M, 6 F), TH-DIO-ChR2 + DRD1 Antagonist: *n* = 9 (4 M, 5 F)] [vGAT-DIO-mCherry *n* = 12 (6 M, 6 F), vGAT-DIO-ChR2 *n* = 13 (5 M, 8 F)]. [Extinction Recall: Three-way ANOVA: DRD1 Block × Opto × Sex. DRD1 Block × Opto × Sex Interaction: *p* = 0.9538, Opto × Sex interaction: *p* = 0.1765, DRD1 Block × Opto Interaction: *p* = 0.0188)]. We did not find any sex differences in our data and have reported our data split by sex in Supplementary Figs. 13 and 14.

## Discussion

Our data reveal how calibration and functional segregation of fear inhibition is achieved by cellular heterogeneity in the ZI and provide functional evidence for the ZI → RE incerto-thalamic pathway being a nuanced modulator of fear.

Aberrant activity in the thalamus has been reported in individuals suffering from PTSD and Generalized Anxiety Disorder [38–44]. In particular, impaired fear inhibition has been associated with increased thalamic activity [45, 46]. Inhibitory control of thalamic nuclei is crucial in shaping appropriate behavioral responses and dysregulation of thalamic inhibition could lead to pathological states that support overgeneralization and persistence of fear memories. One of the major sources of inhibitory control of the thalamus arises from the subthalamic ZI. With our identification and demonstration of the distinct inhibitory pathway arising from the ZI to thalamic RE, we asked whether this pathway contributes to appropriate fear expression, i.e., inhibition of fear responses in safe conditions and suppression of fear responses to cues that were previously associated with threat but are no longer dangerous. Using cell-type-specific and projection-specific optogenetic strategy, we found that stimulation of GABAergic projection fibers from ZI-RE abolished fear generalization in animals trained under high threat conditions. These data build on our previous report [19] that stimulating GABAergic cells in the ZI reduced fear generalization in animals trained under high threat conditions. Notably, the observed blockade of fear generalization was due to decreased fear responses expressed specifically towards the CS− but not the CS+. Furthermore, while stimulation of this inhibitory pathway does not alter fear responses during extinction training, it resulted in enhanced extinction recall, one day later. Prior literature has demonstrated roles for ZI-located GABAergic cells [18, 19, 21], and the RE in fear generalization and extinction learning [16, 17, 31]. Our data provide evidence of a functional link between these two regions and suggest that the ZI → RE GABAergic pathway can play an important role not only in suppressing fear generalization but also in the facilitation of extinction recall.

While GABAergic cells are the predominant cell population in the ZI, the ZI is chemoarchitecturally heterogeneous [22, 47]. Among this cellular heterogeneity, the A13 cluster of dopaminergic cells was an extremely attractive sub-population of cells to consider in the context of dopamine’s rich history in learning and memory [48], and recent reports of the influences of A10 dopaminergic neurons in the VTA on fear generalization as well as extinction learning [24–26]. Second, published literature [36, 37] and our reported data demonstrate that A13 dopaminergic cells project to the RE (Fig. 3). As would be expected, these projections from a small subset of cells in the ZI are not as dense as the projections from the bulk of ZI-located GABAergic cells to the RE that we visualized and manipulated in vGAT-CRE mice. However, our work has revealed the functional relevance of these ZI → RE A13 projections using a combination of optogenetics and pharmacology. Using a targeted optogenetic approach, we found that while stimulation of dopaminergic ZI → RE projections did not suppress fear generalization, extinction recall was facilitated one day after extinction training. Data exist to demonstrate that stimulation of a subset of VTA → lateral habenula neurons that express dopaminergic markers does not result in the release of dopamine and has effects on reward-related behavior that are mediated by GABA-responsive receptors [49]. The co-expression of GABAergic and dopaminergic markers in the ZI might suggest a similar GABA-based and dopamine-independent neuromodulation of extinction learning. However, within the ZI, our data suggest that dopamine action via the D1 dopamine receptor was responsible for the facilitation of extinction recall observed after stimulation of A13 ZI → RE terminals during extinction training. Future analyses could seek to corroborate that it was indeed dopamine that is responsible for the aforementioned facilitation of extinction recall after stimulation of A13 ZI → RE projections and not GABAergic signaling by demonstrating that stimulation of A13 ZI → RE terminals during extinction training resulted in dopamine release in the RE, and by demonstrating continued facilitation of extinction recall after stimulation of A13 ZI → RE terminals even after administration of a GABAergic antagonist during extinction training. Our focus on DRD1 was due to its abundant expression in the RE compared to DRD2 (Supplementary Fig. 15). Our data in no way diminish the role of DRD2 in extinction learning and future studies would do well to probe how extinction learning is influenced by A13 projections to DRD2-expressing extinction learning-related neuroanatomy outside the RE. On a related note, given that the A13 cells co-express GABA and therefore make up a subset of the neurons activated in Fig. 1E, it stands to reason that the enhancement of extinction recall observed after stimulation of the GABAergic ZI → RE cell terminals (Fig. 1E) may be driven by dopamine signaling and it would be interesting for future experiments to test whether facilitation of extinction recall observed after stimulation of GABAergic ZI → RE terminals is blocked by a D1 dopamine receptor antagonist.

Recent studies suggest that A10 dopaminergic cells modulate extinction learning by signaling negative prediction error [24–26]. However, single-unit recordings of GABAergic cells in ZI during extinction training did not indicate a temporal connection between firing activity and the foot-shock omission [18]. These data would argue against the influence of A13 dopaminergic cells in encoding negative prediction error. However, to definitively address the involvement of A13 cells in prediction error signaling, optogenetic manipulation should coincide only with the expected US omission period (0.5 s). Optogenetic stimulation in our experiments coincided with the entire 30 s of the CS+ or CS− presentations as has been used in the previously published literature that has used optogenetic stimulation to interrogate cue-based learning and memory [13, 18, 21, 50]. The 30-s interval between extinction trials should allow for recovery of ChR2 from desensitization after laser stimulation. Future experiments would do well to pulse the laser for shorter time durations throughout the 30 s CS presentations and include longer inter-trial intervals.

In viewing our extinction training data, it bears noting that the vGAT-CRE and TH-CRE animals showed different extinction profiles, potentially due to background strain differences and the stress associated with i.p. injections to the TH-CRE animals. High levels of freezing during the test of extinction recall even after extinction training are a function of the high foot-shock intensity used during the training session [32, 34]. Additional proof of the enhancement of extinction recall after stimulation of both, GABAergic and A13 ZI → RE projections, comes from the observations that freezing levels to the CS+ on the day of testing for extinction recall in these groups were significantly lower (approximately 49%) in comparison to all the other groups that show relatively high freezing levels to the CS+ (approximately 64%). One could interpret not seeing the good acquisition of extinction during extinction training in the TH-DIO-mCherry + VEH, TH-DIO-mCherry + DRD1 Antagonist, and TH-DIO-ChR2 + DRD1 antagonist groups as being the explanation for not seeing good recall in these groups one day later. While possible, the TH-DIO-ChR2 + VEH animals showing better extinction recall one day after extinction training also did not show acquisition of extinction on the day of extinction training like the other groups. This specificity further suggests to us that stimulating A13 ZI → RE projections during extinction training enhances extinction recall one day later. To complement these projection-specific data and further our case for stimulation ZI-located A13 cells during extinction training being able to facilitate extinction recall, we used chemogenetics to stimulate the activity of ZI-located A13 cells only during the time of extinction training via CNO administration and then tested for extinction recall, one day later (Supplementary Fig. 16). As shown in Supplementary Fig. 16C, stimulation of ZI-located A13 cells only during the time of extinction training did not influence the recall of previously acquired fear memory (early) and resulted in within-session extinction (late) comparable to the control group. When tested for extinction recall, one day later, animals in which the ZI-located A13 cells had been stimulated using chemogenetics only during the time of extinction training showed better extinction recall compared to the control group (Supplementary Fig. 16D). Albeit not projection-specific, these data provide further evidence for the ability of ZI-located A13 cells to modulate extinction learning.

Our in vitro current-clamp recordings establish the presence of functional connections between the ZI and the RE. Technically, light stimulation of axon terminals has been shown to antidromically activate cell soma [51–53]. Here we could not rule out the possibility that photostimulation of RE-located axon terminals originating from cells in the ZI may cause antidromic activation of soma of these neurons in the ZI. The subsequent release of neurotransmitters in non-RE regions downstream of ZI-located cells, in theory, could then be responsible for the reported effects on fear generalization and extinction learning. While we acknowledge this possibility, to our knowledge, there is no clear evidence to suggest that antidromically activated neurons could induce secondary neurotransmitter release at other axon collaterals in secondary non-specific regions to affect neural function and behavior.

Previous studies have demonstrated that the RE is important for maintaining the specificity, generalization, and extinction of fear memory representations [15–17, 29, 31]. The general consensus from these studies is that inactivation of the RE results in fear generalization and impaired extinction recall. Our findings in the current study appear contradictory. However, key methodological differences and our collective lack of appreciation for cell types within the RE might explain the discrepancy in results. First, direct prolonged inactivation of the RE using muscimol or tetanus toxin could alter behavior differently compared to brief ZI-mediated optogenetic inhibition of RE. Second, inactivation of the entire RE using muscimol and tetanus toxin is broader than our inhibition of only the RE neurons that are post-synaptic partners of GABAergic and dopaminergic projections from the ZI. To gain a more nuanced appreciation for how cell types in the RE contribute to fear generalization and extinction learning, we will need to identify the neurochemistry of the RE-located post-synaptic partners of the GABAergic and A13 cells in the ZI.

In agreement with previous literature [23, 54–56], our neurochemical data show that A13 dopaminergic cells co-express GABA. Therefore, it is surprising to find that stimulation of ZI → RE dopaminergic terminals enhanced extinction recall but had no effect on fear generalization, while stimulation of ZI → RE GABAergic terminals reduced fear generalization and enhanced extinction recall. These data suggest that not all GABAergic cells in the ZI are created equal in their ability to modulate fear. Our study supports the existence of at least two anatomically overlapping but functionally distinct groups of GABAergic cells in the ZI that contact the RE – the smaller A13 cluster that modulates extinction recall within a larger GABAergic population that modulates both fear generalization and extinction recall. This functional dichotomy could be attributed to differences arising from the incoming afferents to the GABAergic and A13 cells in the ZI, their post-synaptic partners in the RE, or the chemoarchitecture of the GABAergic cells in the ZI. With GABAergic neurons in the ZI being a much larger population of cells that include both, dopaminergic A13 cells and non-dopaminergic cells, our reported data may suggest that GABAergic ZI → RE cells that are not dopaminergic are important for fear generalization and extinction recall while GABAergic ZI → RE cells that are dopaminergic are exclusively important for extinction recall. Such functional heterogeneity of sub-populations of ZI-located GABAergic cells seems reasonable and necessary for a brain region that is now implicated in behaviors that include learning, memory, sleep, eating, pain, and fear expression [18–21, 57–59]. While a small percentage of interneurons have been identified in the primate ZI [60], no data to our knowledge have demonstrated the existence of interneurons in the rodent ZI and our experiments targeted projections of ZI-located cells that terminated in the RE.

Accumulating evidence emphasizes the central influence of incertal circuitry on fear and defensive behaviors. Through efferent innervation of the nucleus RE, periaqueductal gray, superior colliculus, cuneiform nucleus, and the pedunculopontine-tegmental-nucleus [18, 61–63], the ZI is perfectly positioned to integrate information from brain regions that process emotionally salient stimuli and interface with canonical neurocircuitry that play important roles in fear expression and avoidance. Notably, inhibitory inputs from the central amygdala to the ZI have been implicated in the acquisition of fear memories and remote memory retrieval [21], and pharmacological silencing of the prefrontal cortex blunts extinction-associated increases in neuronal firing in the ZI [18]. Therefore, inclusion of the mostly ignored ZI is required to gain a deeper understanding of parallel distributed circuits that control fear generalization and extinction learning. Fear generalization and fear extinction are distinct behavioral dimensions of fear expression, potentially controlled by overlapping yet distinct neural circuits. Therefore, an argument could be made for optogenetic stimulation during the generalization test confounding subsequent extinction training. Optogenetic stimulation (paired with 2× CS−) performed during the generalization test did not interfere with the subsequent extinction training, as evident from the within-session extinction of fear observed in Fig. 2D. Further, extinction is highly cue-specific, and previous studies have shown that repeated exposure to a neutral stimulus does not affect the extinction of fear towards the conditioned stimulus [64, 65].

In summary, the present study reveals an adaptive function of the ZI → RE pathway in facilitating the precision and extinction of fear responses, and consequently suggests the potential of using stimulation of this circuit to remedy deficits in fear inhibition in PTSD. The finding that this pathway can suppress fear generalization toward neutral cues and/or enhance extinction recall, suggests that activity in this pathway might be crucial for calibrating fear. In so doing, our work sheds light on neuroanatomy that ought to be considered in discussions about mitigating debilitating bouts of fear that characterize trauma- and anxiety-related disorders.

## Funding and disclosure

We thank the Veterinary and Animal Care staff in the Yerkes Neuroscience Vivarium for animal husbandry. Funding for this study was provided to BGD by the US National Institute of Health (R01MH120133) which included support of AV and JG. Support to BGD was also provided by the Department of Psychiatry and Behavioral Sciences at Emory University School of Medicine, the Yerkes National Primate Research Center (YNPRC), the Department of Pediatrics at USC Keck School of Medicine, the Division of Research on Children, Youth & Families at Children’s Hospital Los Angeles, and the Developmental Neuroscience and Neurogenetics Program at The Saban Research Institute. AV was also supported by the Emory University Women’s Club Memorial Fellowship Award. LJY and KI were supported by P50MH100023 to LJY. JG’s contributions were also supported by R01MH072908 to LJY. Additional funding was provided to Yerkes National Primate Research Center by Office of Research Infrastructure Programs ODP51OD11132. The authors have no competing financial interests or potential conflicts of interest.

## Supplementary information

Supplemental Material
