# Supplementary material for: Incerto-thalamic modulation of fear via GABA and dopamine
Source: Neuropsychopharmacology. 2021 Apr 16;46(9):1658–68. doi: 10.1038/s41386-021-01006-5 (PMC8280196; doi:10.1038/s41386-021-01006-5)
Supplement: Supplementary file 1 — Supplemental Material [file 41386_2021_1006_MOESM1_ESM.docx]

**Incerto-thalamic modulation of fear via GABA and dopamine.**

**Venkataraman et al.**

**SUPPLEMENTARY FIGURES**


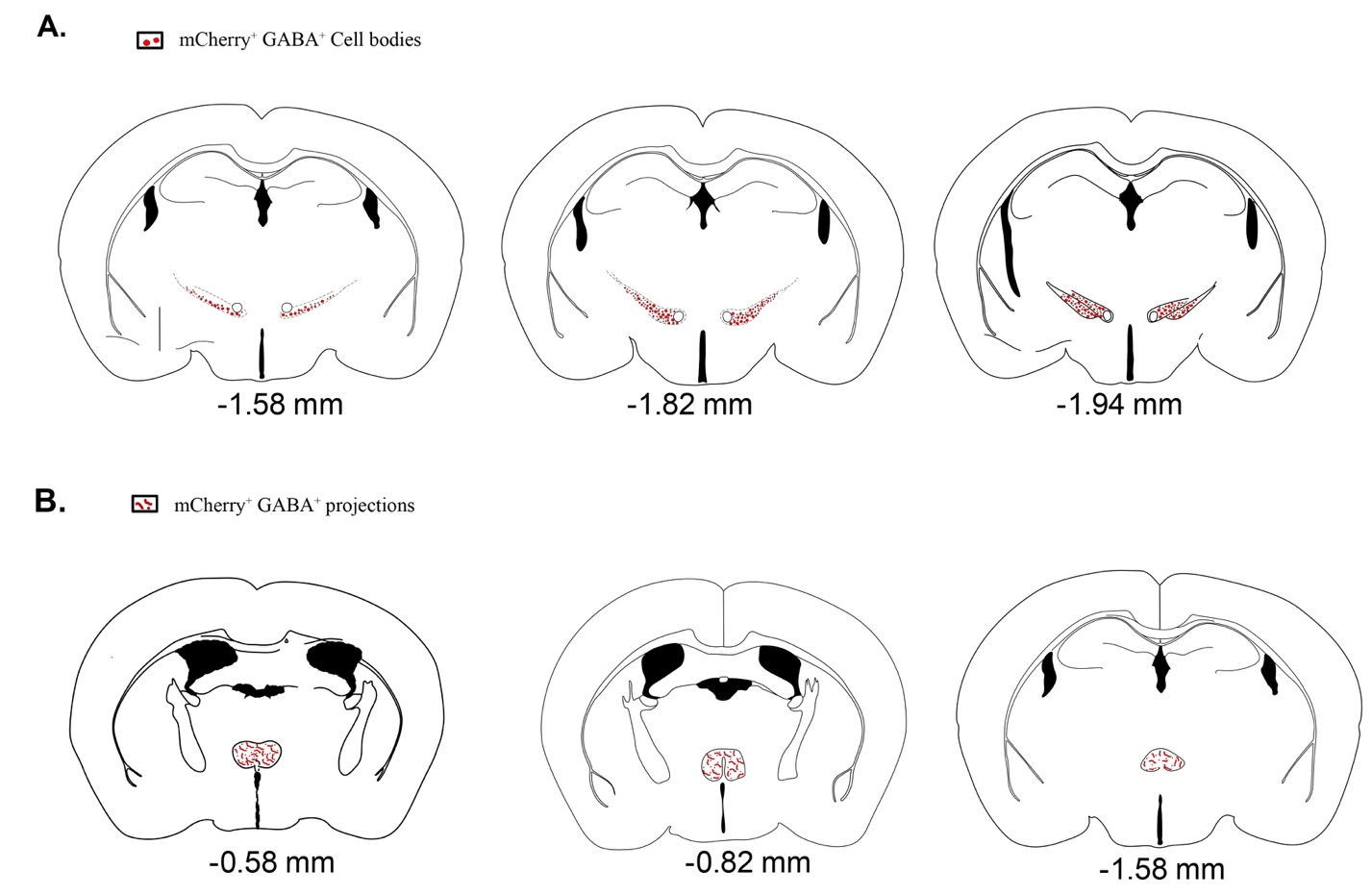


**Supplementary Figure 1: Schematic of GABAergic cell bodies in the ZI and projections in the RE of vGAT-CRE mice.**

**(A)** vGAT-CRE mice were injected with either the control virus (AAV5-EF1α-DIO-mCherry) or Cre-dependent ChannelRhodopsin2 (AAV-EF1α-DIO-ChR2-mCherry) into the ZI at -1.5mm posterior to bregma and viral expression were restricted to the medial portion of the ZI within the coordinates noted in the Methods section. **(B)** The optic fiber was placed above the RE at -0.38 mm posterior to bregma and the mCherry expressing GABAergic projections were visualized in the RE as indicated.

**
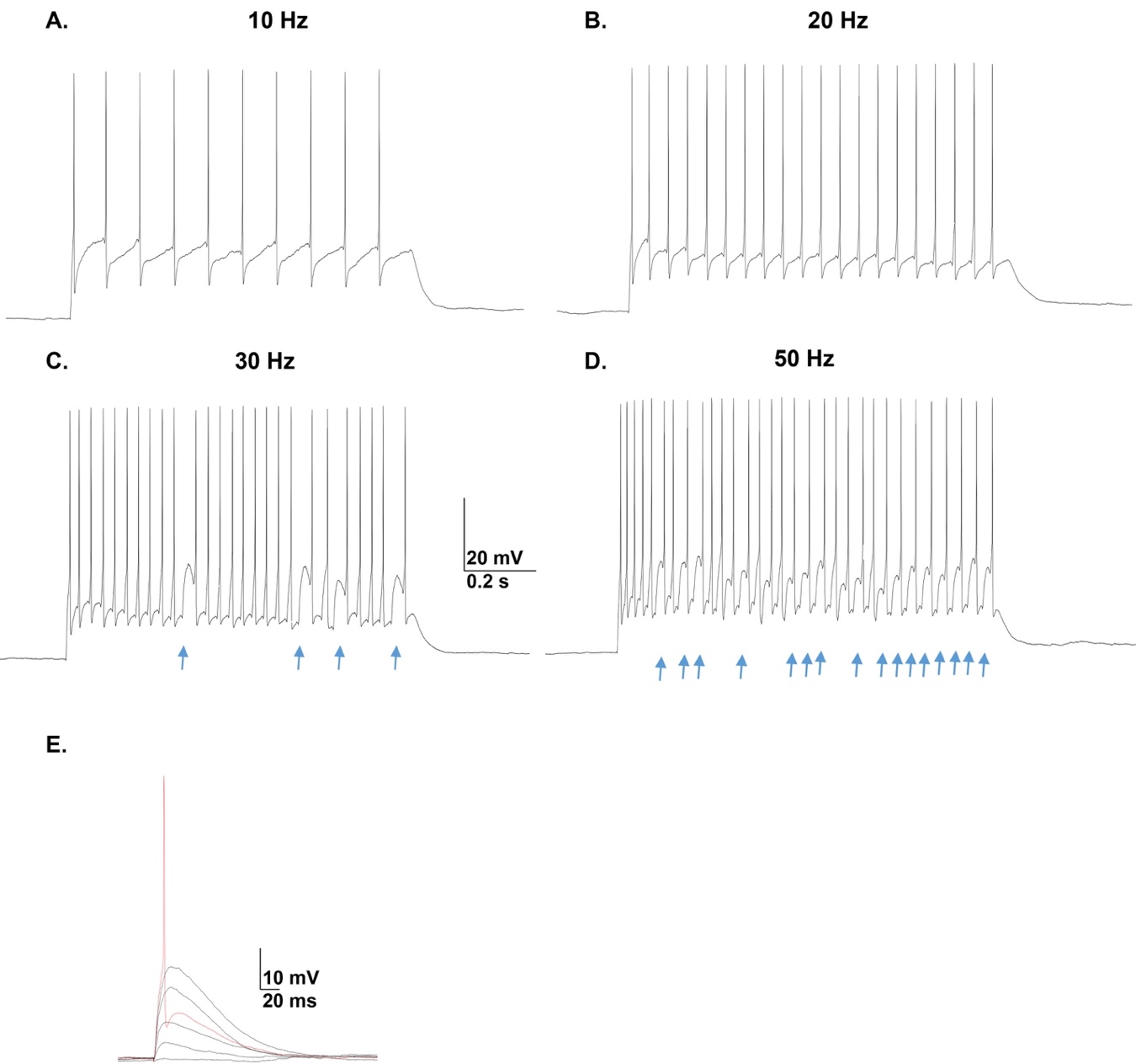
**

**Supplementary Figure 2: Optogenetic control of action potential firing in ZI GABAergic neurons transfected with ChR2 opsin [**[66](#_ENREF_66)**]** Responses of GABAergic neurons in the ZI neurons to light pulse trains of different stimulation frequencies. In response to **(A)** 10 Hz **(B)** and 20 Hz light pulse trains, the ZI neuron fires an action potential reliably following stimulation frequency, while at higher frequencies **(C&D)**, the firing success rate decreased. **(E)** Representative sweeps showing light induced membrane depolarization that increased in amplitude with increases in light intensity (0.4-5.0 mW/mm^2^). An action potential was evoked when threshold was reached. Arrows in C and D denote failures to fire an action potential.


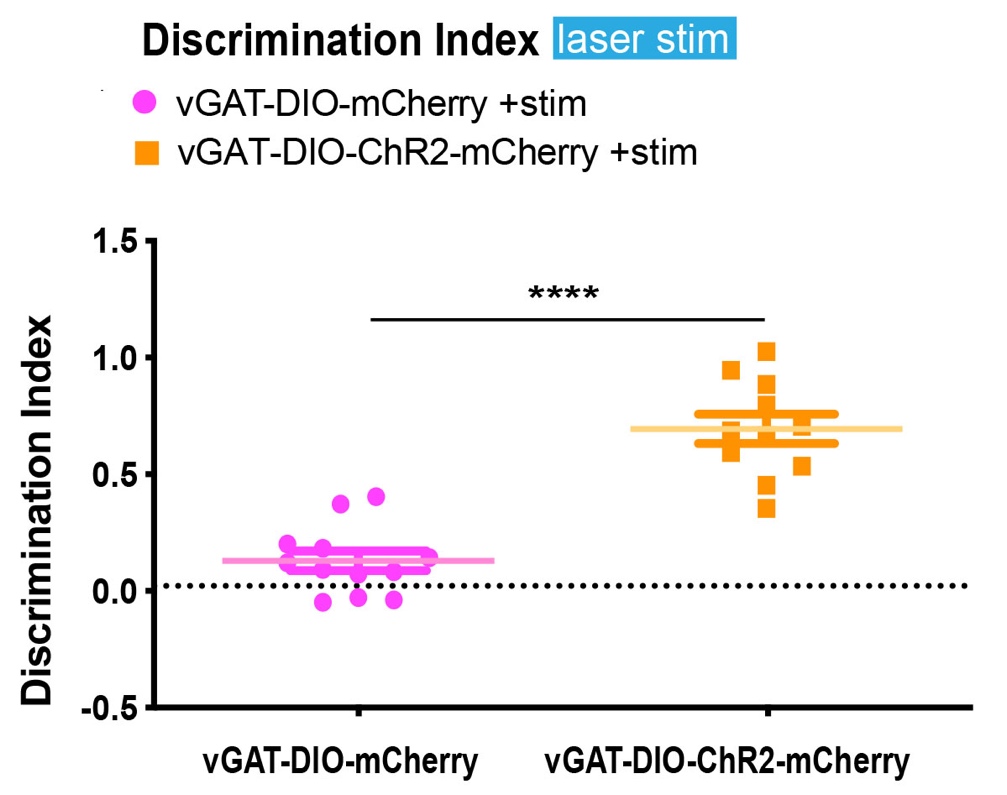


**Supplementary Figure 3: Targeted optogenetic stimulation of ZI🡪RE GABAergic projections enhanced discrimination between CS+ and CS-.** Optogenetic stimulation of ZI-RE GABAergic projections in vGAT-CRE:DIO-ChR2-mCherry+stim animals produced better fear discrimination compared to vGAT-CRE:DIO-mCherry+stim control animals (p < 0.0001, t = 7.601, df = 21). Discrimination index (DI) was calculated using the following formula:

DI = freezing to CS^+^ – freezing to CS^-^

freezing to CS^+^ + freezing to CS^-^


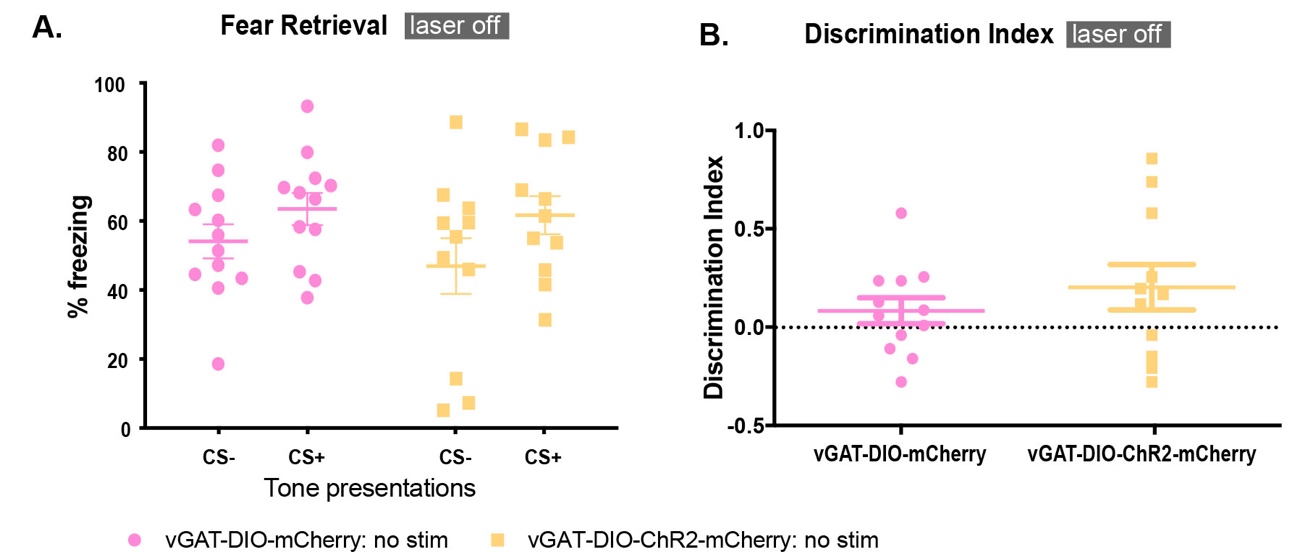


**Supplementary Figure 4: High intensity auditory fear conditioning produced fear generalization in the absence of targeted optogenetic stimulation of ZI🡪RE GABAergic projections.** 24 hrs after high-intensity auditory fear conditioning, in the absence of optogenetic activation of ZI-RE GABAergic projections, both vGAT-CRE:DIO-ChR2-mCherry and vGAT-CRE:DIO-mCherry groups showed **(A)** similarly high levels of freezing to the CS+ and CS- and **(B)** enhanced fear generalization as evidenced by their low discrimination indices. Discrimination index (DI) was calculated using the following formula:

DI = freezing to CS^+^ – freezing to CS^-^

freezing to CS^+^ + freezing to CS^-^

**
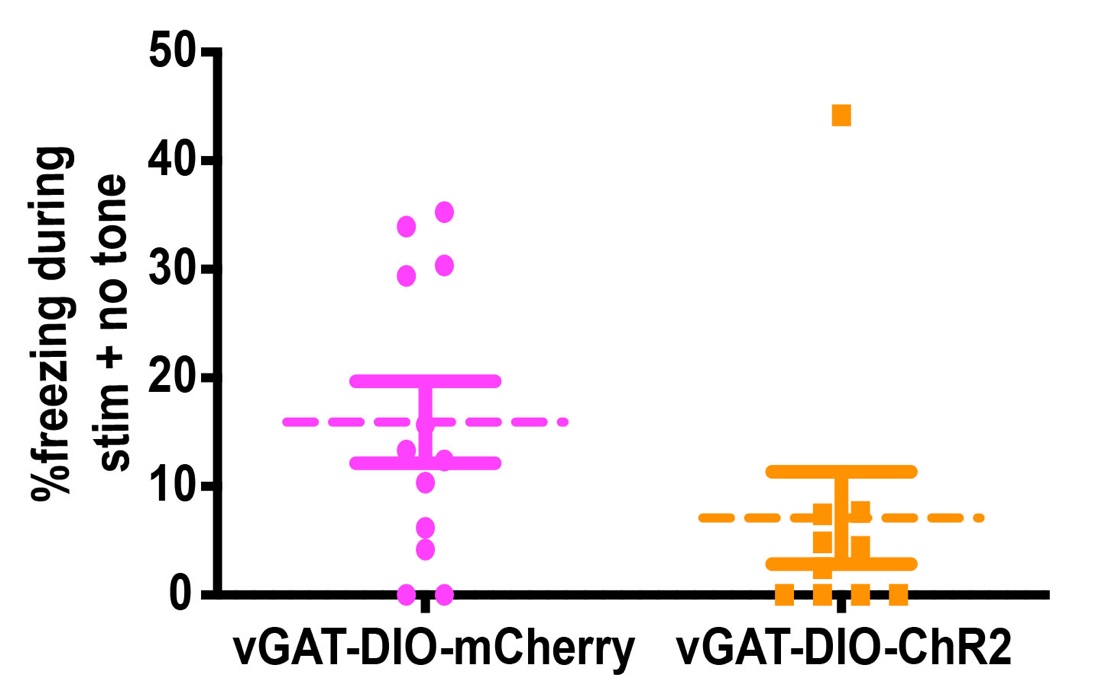
**

**Supplementary Figure 5: Optogenetic activation of ZI🡪RE GABAergic projections does not produce non-specific changes in freezing responses.** Laser stimulation of GABAergic projections from ZI to RE on testing day (in the absence of tones) did not produce any significant differences in freezing between the two groups (vGAT-DIO-mCherry, vGAT-DIO-ChR2). Data represented as Mean ± S.E.M.


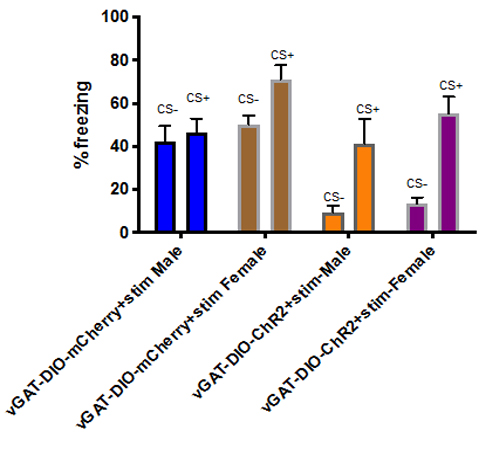


**Supplementary Figure 6: Targeted optogenetic stimulation of ZI🡪RE GABAergic projections reduced fear generalization in male and female mice.** No significant differences were observed in freezing levels between sexes, with optogenetic stimulation of ZI-RE GABAergic projections during fear retrieval. Data represented as Mean ± S.E.M.

**
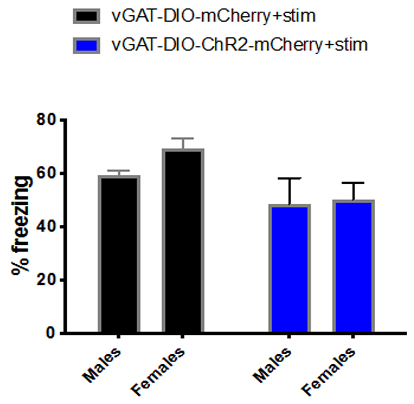
**

**Supplementary Figure 7: Targeted optogenetic stimulation of ZI🡪RE GABAergic projections enhanced extinction recall in male and female mice.** No significant differences were observed in freezing levels during extinction recall between sexes, with optogenetic stimulation of ZI-RE GABAergic projections. Data represented as Mean ± S.E.M.


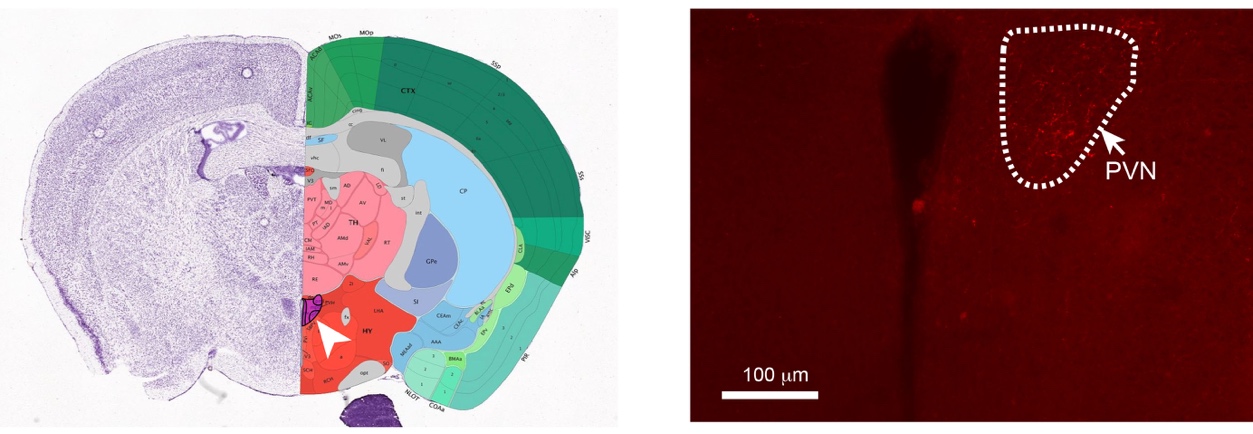


**Supplementary Figure 8: Validation of projections of A13 dopaminergic cells.** Projections visualized in the paraventricular nucleus of the hypothalamus (PVN) after infusion of AAV-DIO-mCherry into the ZI of TH-CRE mice.


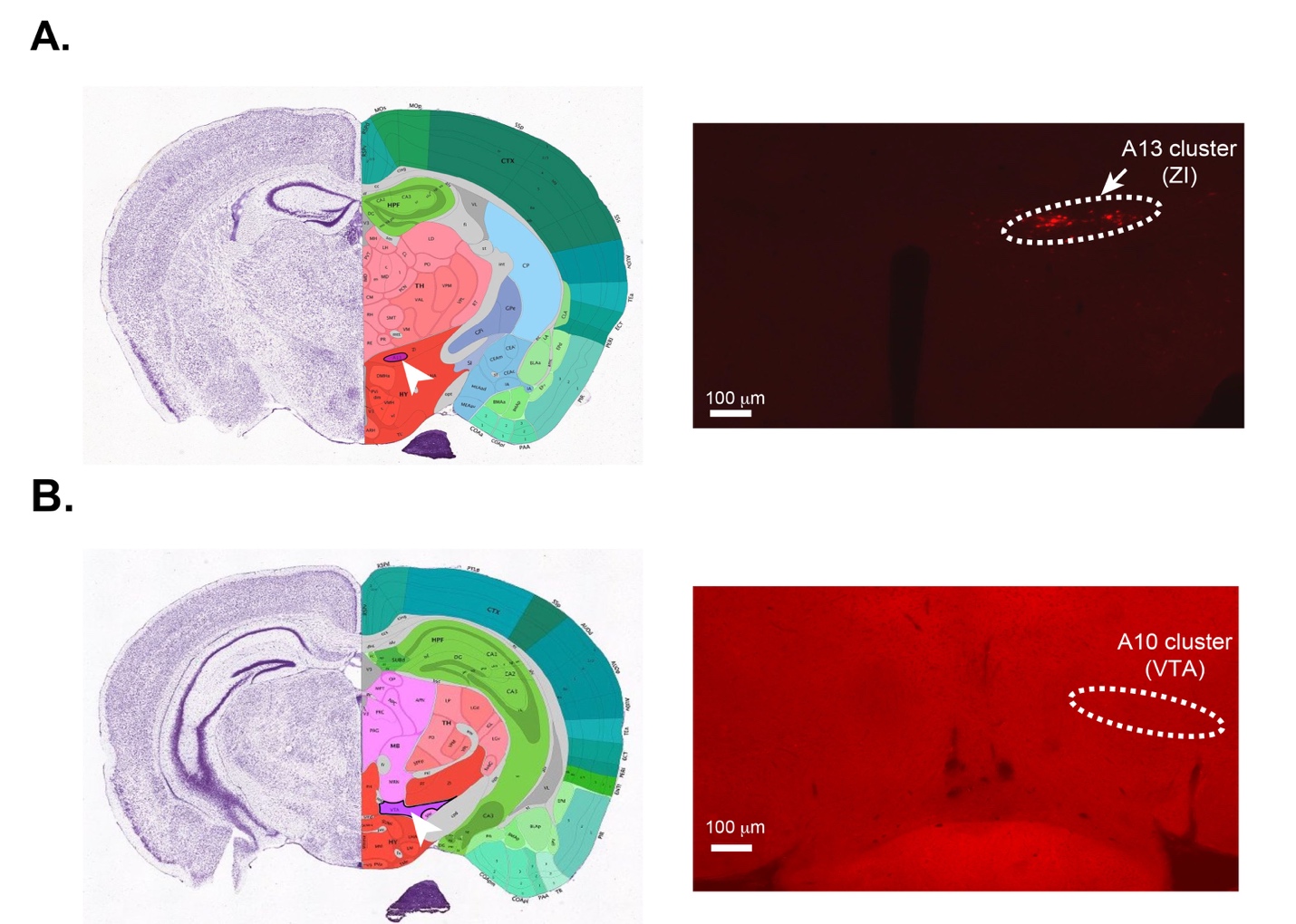


**Supplementary Figure 9: Specificity of targeting A13 dopaminergic cells with virus infusions while leaving A10 cells intact. (A)** A13 cells in the ZI visualized as expressing mCherry after infusion of AAV-DIO-mCherry into the ZI of TH-CRE mice. **(B)** A10 cells in the VTA of same animal do not express mCherrry indicating specificity of targeting A13 cells. Section with VTA over-exposed intentionally to indicate no staining of A10 cells.


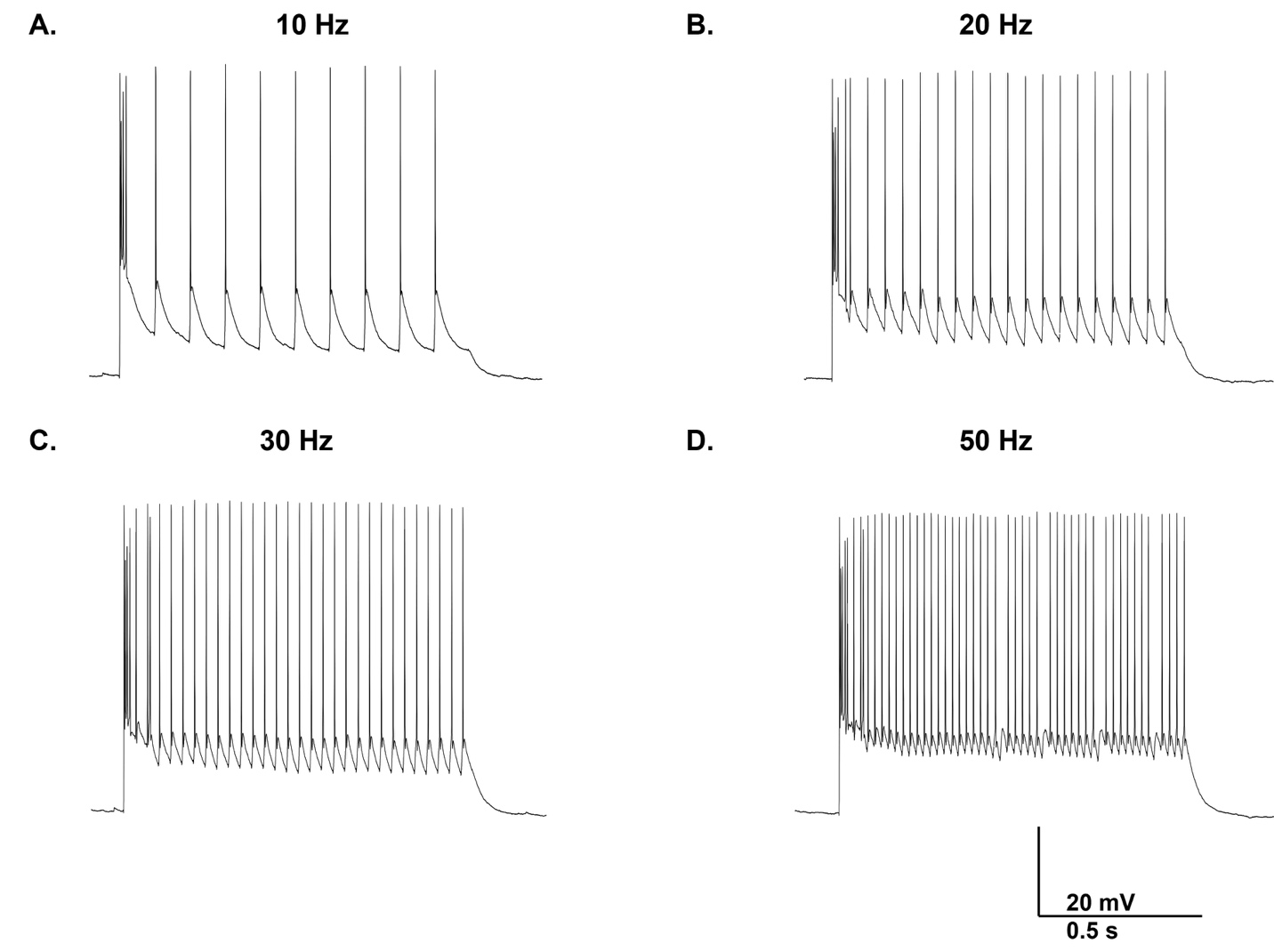


**Supplementary Figure 10: Optogenetic control of action potential firing in ZI dopaminergic neurons transfected with ChR2 opsin.** Responses of ChannelRhodopsin-infected dopaminergic (TH-positive) neurons in the ZI to light pulse trains of different stimulation frequencies. In response to **(A)** 10 Hz, **(B)** 20 Hz, and **(C)** 30 Hz light pulse trains, A13 cells in the ZI fire action potentials reliably following stimulation frequency, while at higher frequencies **(D)**, the firing success rate decreased.

**Supplementary Figure 11: Targeted optogenetic stimulation of ZI🡪RE A13 dopaminergic projections does not enhance discrimination between CS+ and CS-.** High intensity foot-shock training resulted in poor discrimination between CS+ and CS- in TH-DIO-mCherry+stim animals. Optogenetic stimulation of ZI🡪RE A13 dopaminergic projections in TH-DIO-ChR2+stim animals did not rescue this poor discrimination. Discrimination index (DI) was calculated using the following formula:

DI = freezing to CS^+^ – freezing to CS^-^

freezing to CS^+^ + freezing to CS^-^

**
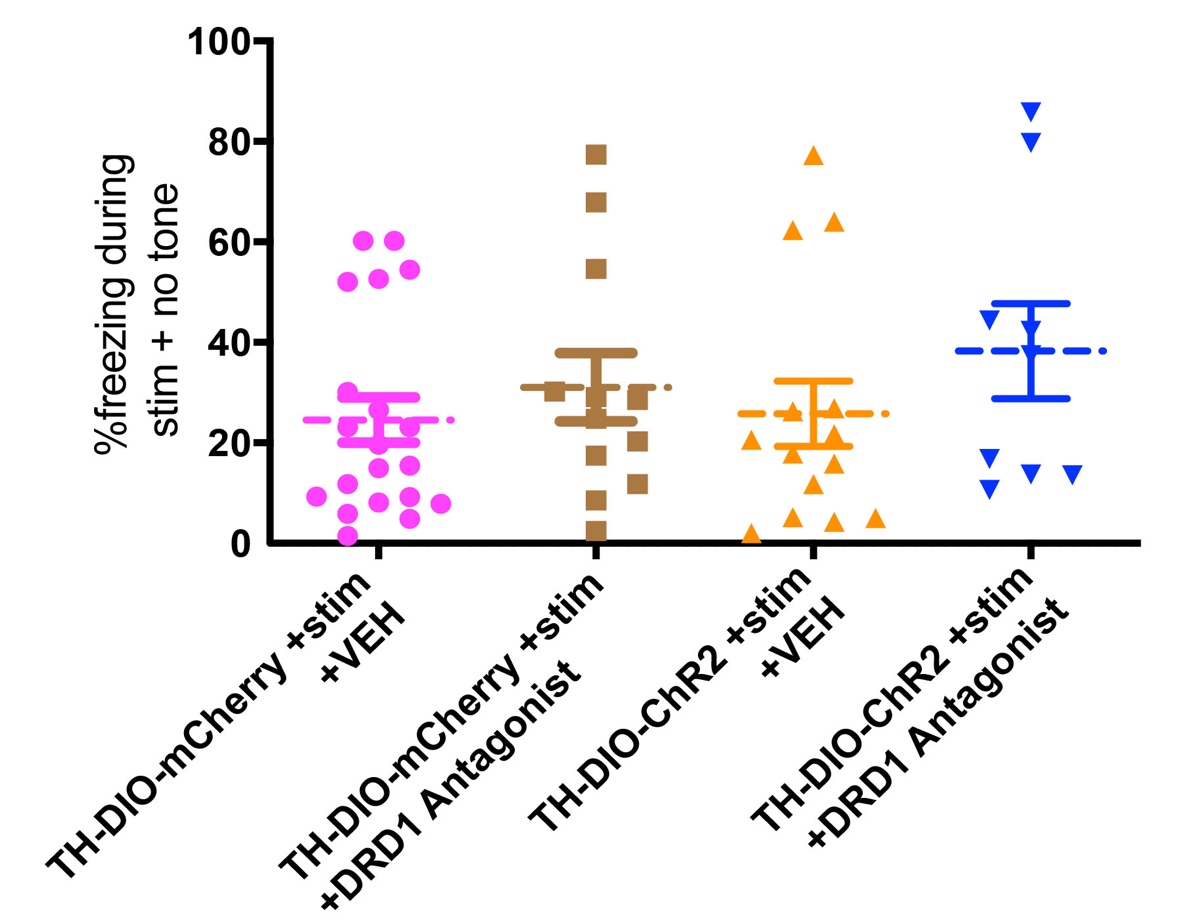
**

**Supplementary Figure 12: Optogenetic activation of ZI🡪RE dopaminergic projections and pre-treatment with DRD1 Antagonist does not produce non-specific changes in freezing responses.** Laser stimulation of dopaminergic projections from ZI to RE on testing day (in the absence of tones) did not produce any significant differences in freezing between groups infected with Channelrhodopsin or Control and pre-treated with DRD1 Antagonist or Vehicle. (TH-DIO-mCherry+stim+Vehicle, TH-DIO-mCherry+stim+DRD1 Antagonist, TH-DIO-ChR2-mCherry+stim+Vehicle, TH-DIO-ChR2-mCherry+stim+DRD1 Antagonist). Data represented as Mean ± S.E.M.

**
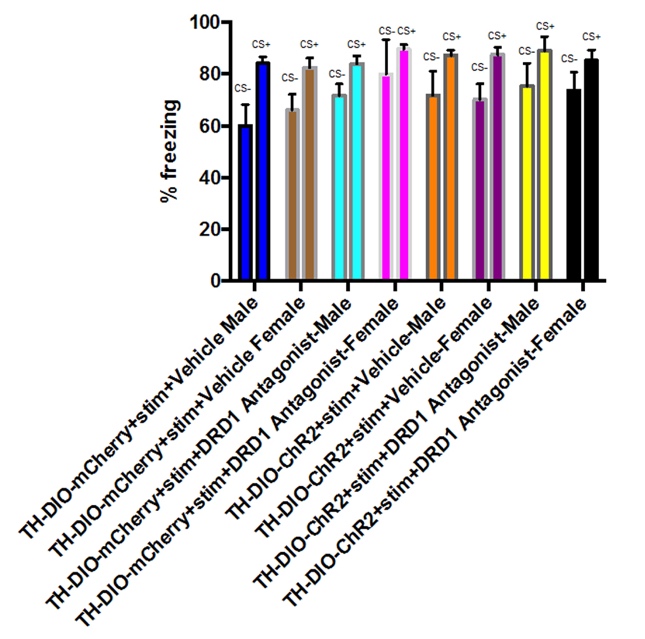
**

**Supplementary Figure 13: Targeted optogenetic stimulation of ZI🡪RE A13 dopaminergic projections did not reduce fear generalization in either male or female mice.** No significant differences were observed in freezing levels between sexes, with optogenetic stimulation of ZI-RE A13 dopaminergic projections during fear retrieval. Data represented as Mean ± S.E.M.

**
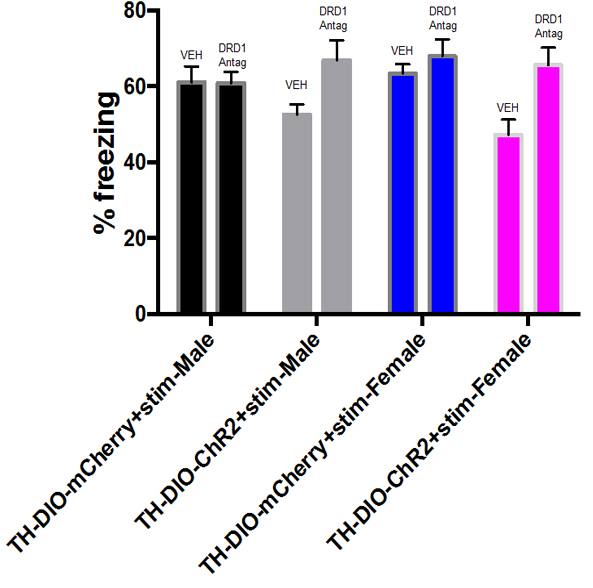
**

**Supplementary Figure 14: Targeted optogenetic stimulation of ZI🡪RE A13 dopaminergic projections enhanced extinction recall in male and female mice in a DRD1-dependent manner.** No significant differences were observed in freezing levels during extinction recall between sexes, with optogenetic stimulation of ZI-RE A13 dopaminergic projections. Data represented as Mean ± S.E.M.


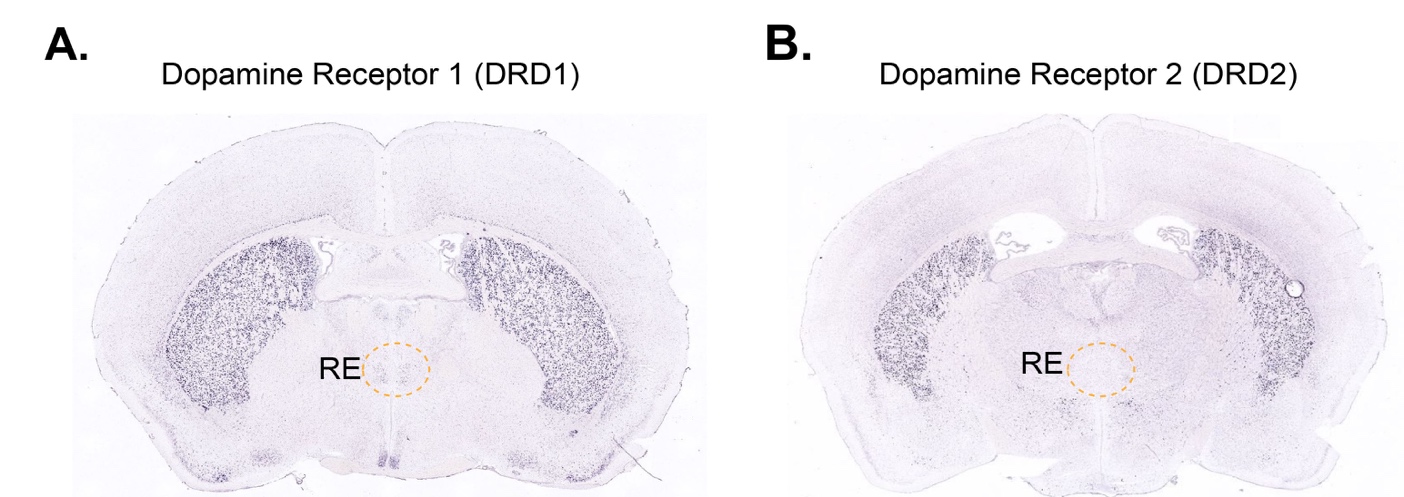


**Supplementary Figure 15: Expression of dopamine receptors in ZI.** Allen Brain Atlas *in situ* hybridization experiments reveal expression of DRD1 in the RE **(A)**, while DRD2 expression appears to be undetectable **(B)**.


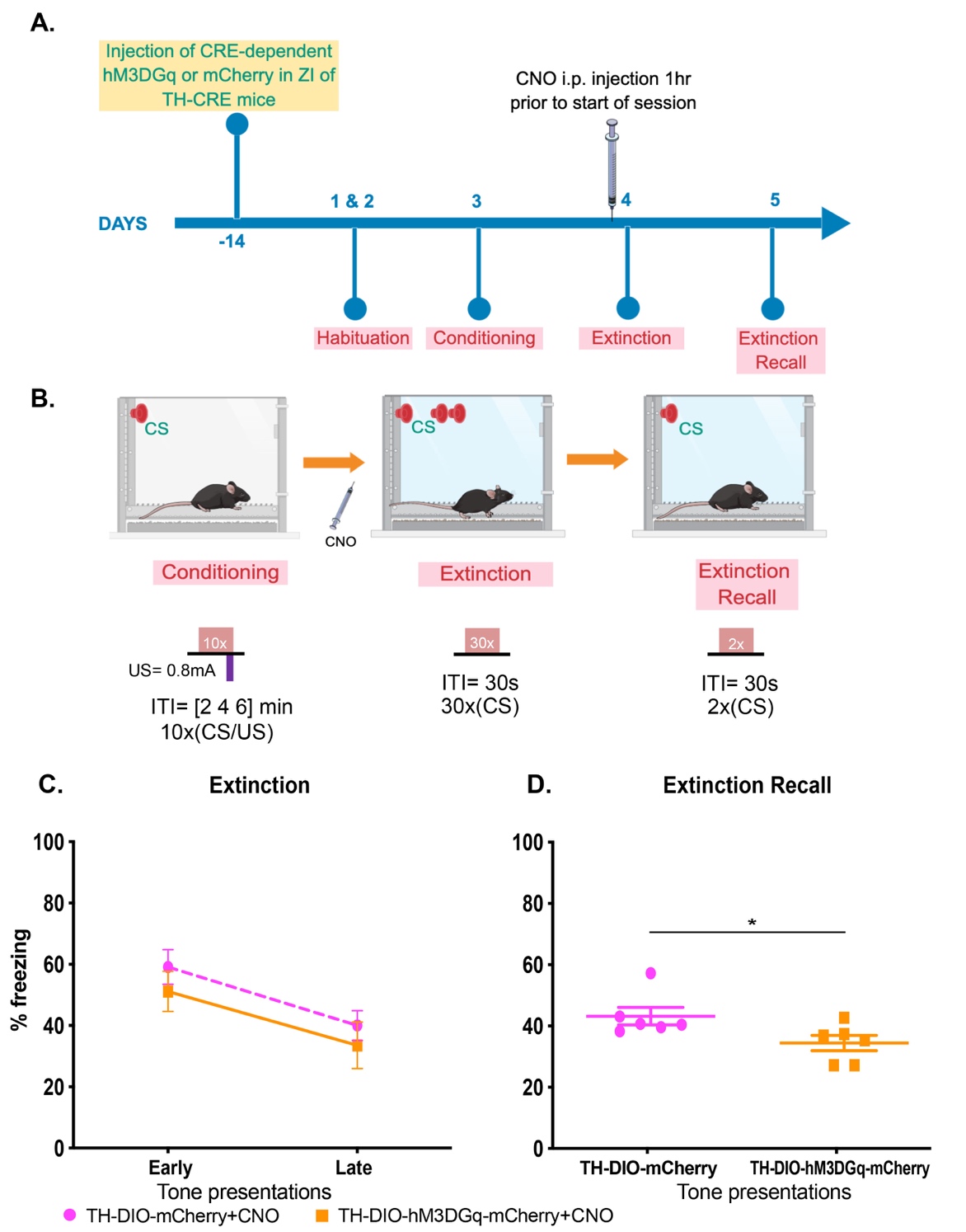


**Supplementary Figure 16: Targeted chemogenetic stimulation of A13 dopaminergic cells in the ZI during extinction training enhances extinction recall. (A)** Experimental design: TH-CRE animals received intracranial injections of CRE-dependent control (DIO-mCherry) or hM3DGq (DIO-hM3DGq) virus in the ZI. After 2 weeks, animals were first habituated and then fear conditioned to tones using high shock intensities. One day later, animals were first injected with clozapine-N-oxide (1 mg/kg i.p. in 2% DMSO in 0.9% saline) and put through an extinction session, one hour later. The following day, animals were tested for extinction recall. **(B)**  Outline of the high-intensity auditory fear conditioning protocol used in the study. On training day, both control and treatment groups of mice received CS+ tone presentations paired with 0.8mA foot-shocks (high threat intensity) and unpaired CS- tone presentations. On extinction training day, animals were injected with CNO and then one hour later received repeated presentations of CS+ tones as part of the extinction training session. One day later, fear responses of the animals to the CS+ tones were tested. **(C)** TH-DIO-hM3DGq-mCherry+CNO animals in which A13 dopaminergic cells in the ZI were chemogenetically stimulated during extinction training showed extinction of fear to the CS+ that was similar to the control TH-DIO-mCherry+CNO controls. **(D)** When tested for extinction recall one day after extinction training, TH-DIO-hM3DGq-mCherry+CNO animals in which the A13 dopaminergic cells in the ZI had been chemogenetically stimulated during extinction training showed a significant decrease in fear response to CS+ compared to controls (TH-DIO-mCherry+CNO) (t = 2.304, df = 10). TH-DIO-mCherry+CNO n = 6, TH-DIO-hM3DGq-mCherry+CNO n = 6. * p< 0.05. Data represented as Mean ± S.E.M.
